# Insufficient production of IL-10 from M2 macrophages impairs *in vitro* endothelial progenitor cell differentiation in patients with Moyamoya disease

**DOI:** 10.1038/s41598-019-53114-4

**Published:** 2019-11-14

**Authors:** Eiichiro Nagata, Haruchika Masuda, Taira Nakayama, Shizuka Netsu, Hiroko Yuzawa, Natsuko Fujii, Saori Kohara, Takatoshi Sorimachi, Takahiro Osada, Ryoko Imazeki, Mitsunori Matsumae, Takayuki Asahara, Shunya Takizawa

**Affiliations:** 1 Department of Neurology, Tokai University School of Medicine, Isehara, Japan; 2 Department of Physiology, Tokai University School of Medicine, Isehara, Japan; 3 Department of Neurosurgery, Tokai University School of Medicine, Isehara, Japan; 4 Department of Basic Clinical Science, Division of Regenerative Medicine, Tokai University School of Medicine, Isehara, Japan

**Keywords:** Stroke, Cell signalling

## Abstract

Moyamoya disease (MMD) is well known to be caused by insufficient cerebral vascular formation. However, the essential pathogenesis has not yet been identified. Using our recently developed technique of generating vasculogenic and anti-inflammatory cultures, we investigated endothelial progenitor cell (EPC) expansion and differentiation under the cytokine milieu generated by the peripheral blood mononuclear cells (PBMNCs) of the operated and non-operated MMD patients. EPC colony forming assay of the cultured PBMNCs disclosed the decline of the definitive EPC colony numbers in the both MMD patients. The level of interleukin-10 (IL-10) was lower in secretory cytokines from the cultured PBMNCs of MMD patients than that in that of controls using a cytometric bead array. The addition of human recombinant IL-10 to PBMNCs cultured from MMD patients restored the EPC colony forming potential of MMD PBMNCs. Following phorbol myristate acetate stimulation of the cultured PBMNCs, flow cytometry revealed a decrease in intracellular IL-10 storage in the main cell populations of the PBMNCs cultured from MMD patients relative to those cultured from controls. The present data provide the expected mechanism of vascular malformation in MMD pathogenesis originated from the insufficient production of IL-10 secreting cells from PBMNCs fostering EPC expansion and differentiation.

## Introduction

Moyamoya disease (MMD) is an idiopathic, intracranial vasculopathy that reveals stenosis of the bilateral terminal internal carotid arteries and a tangled network of basal collaterals. Recurrent stroke occurs among children and adolescents with MMD. Cerebral infarction mainly occurs in children and cerebral haemorrhage occurs in adolescents^1^. While the predominance of MMD amongst East Asian population suggests that its genetic origin, however, recent discovery of a disease-linked genetic variation at the RNF213 locus suggests that the variant may also be associated with other steno-occlusive diseases, such as intracranial arterial stenosis, pulmonary hypertension, and coronary artery disease. Therefore, the pathogenesis of MMD still remains elusive^2–5^.

Previous studies have implicated vasculogenic cytokines in the pathophysiology of MMD^6,7^. Notably, basic fibroblast growth factor (bFGF) and its receptor are highly expressed in the vessels of patients with MMD^5^. Elevated plasma levels of vascular endothelial growth factor (VEGF) and platelet-derived growth factor (PDGF) have also been reported, suggesting that the pathology of MMD involves the recruitment of cellular components by humoral and/or paracrine factors and that cytokines are significantly related to the pathophysiology of MMD^7,8^. However, cytokines, such as VEGF and PDGF, do not primarily cause MMD.

Earlier, studies have shown that circulating endothelial progenitor cells (EPCs) derived from bone marrow contribute to postnatal physiological and pathological neovascularization^9,10^ confirming their role in vasculogenesis. EPCs were first isolated from the peripheral blood of adults in 1997^9^. Circulating EPCs derived from the bone marrow were shown to contribute to postnatal physiological and pathological neovascularisation^10,11^.

In order to obtain EPC-enriched cell populations and investigate the pathophysiology of MMD, including the role of cytokines in MMD, we developed a method to obtain quality- and quantity-controlled culture of unfractionated mononuclear cells (MNCs)^12–14^ in the presence of human recombinant stem cell factor (SCF), thrombopoietin, Flt-3 ligand, VEGF, and interleukin-6 (IL-6). This method is simple and safe that can be used to expand EPCs and activate anti-inflammatory and angiogenic monocytes/macrophages and helper T lymphocytes; these properties enable the delivery of various protective and proangiogenic cytokines and growth factors, such as interleukin-8, IL-10, VEGF and others^12–14^.

This study investigates the relationship between expansion and differentiation ability of EPCs and co-existing monocyte/macrophage-produced cytokines in patients with MMD by using an anti-inflammatory and vasculogenic culture milieu^9,10^.

## Results

### Colony-forming EPCs of patients with MMD did not increase in the cultured peripheral blood mononuclear cells (PBMNCs)

**Figure 1:**
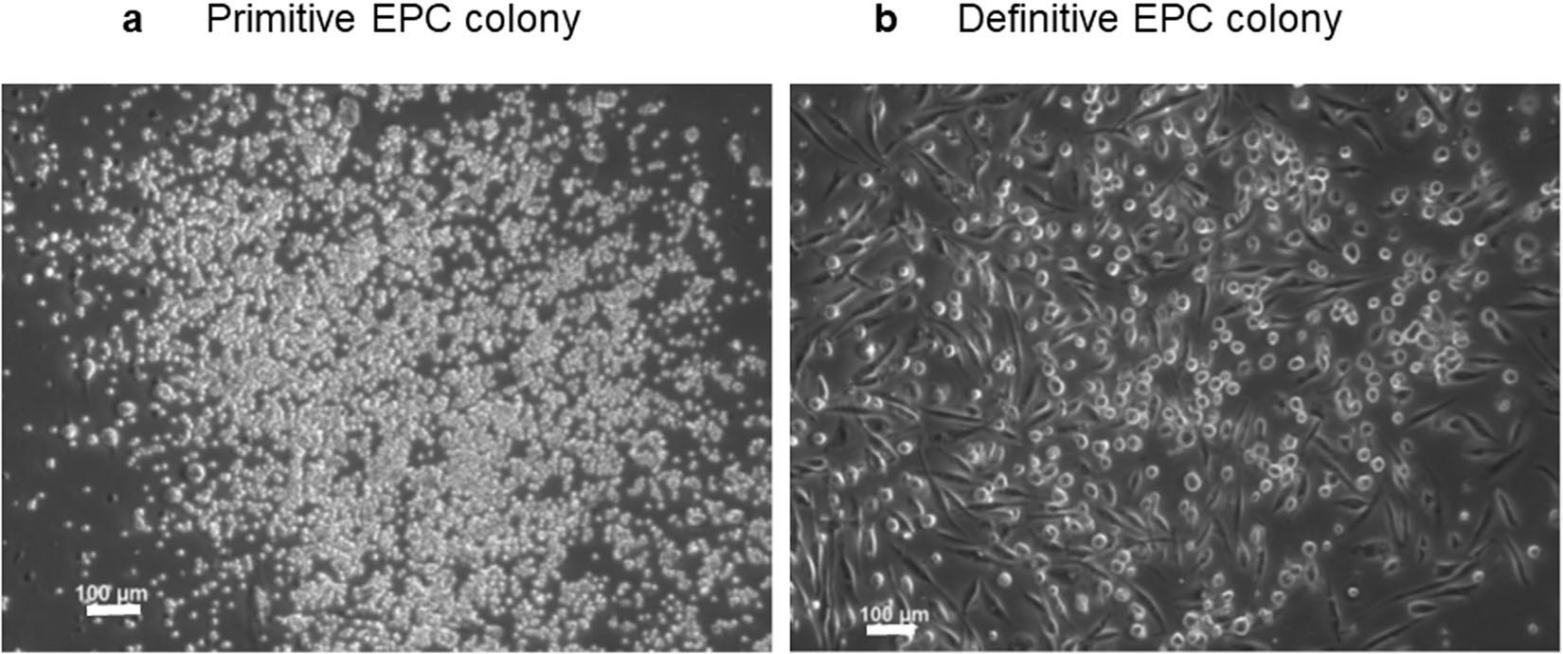
Representative pictures of (**a**) primitive and (**b**) definitive EPC colonies. Scale bar, 100 μm. Primitive EPCs formed small cell colonies. It has poor differentiation potential and expansive ability. On the other hand, definitive EPCs formed large spindle-like cell colonioes. It has differentiation potential and expansive ability.

**Figure 2:**
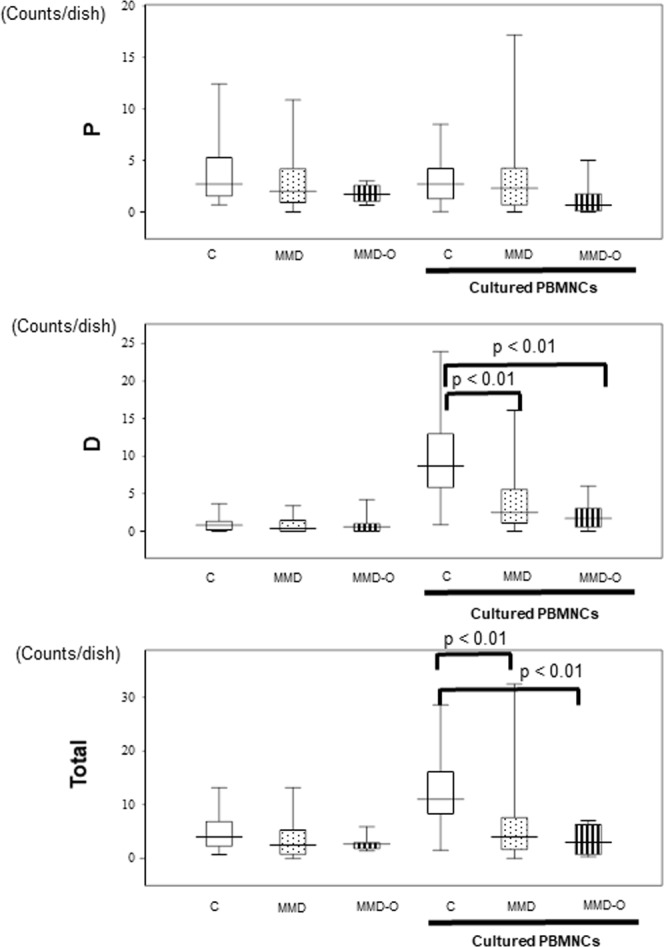
Colony forming assay of freshly isolated and cultured samples derived from patients with MMD, MMD-O, and controls. The number of definitive EPCs increased in the PBMNCs cultured from controls (n = 23) but remained the same in those cultured from patients with MMD (n = 23) or MMD-O (n = 7). With regards to primitive EPC colonies, there was no significant difference between controls and MMD or MMD-O in both PBMNCs and cultured PBMNCs. The figure was generated by plotting values using a box-and-whisker plot. C, control; MMD, Moyamoya disease; MMD-O, patients with MMD who underwent superficial temporal artery to middle cerebral artery operations; PBMNCs, peripheral blood mononuclear cells. P: primitive EPC colonies; D: definitive EPC colonies; count/dish: 35 mm dish was used.

Microscopic results revealed that definitive EPCs forming colonies consisted of large spindle-like cells, were more commonly observed in the PBMNCs cultured from controls (8.70/dish) than in those obtained from healthy controls (0.80/dish) (Fig. 1). The number of colony-forming definitive cells in the PBMNCs cultured from patients with MMD (2.50/dish) or MMD (1.70/dish) who underwent superficial temporal artery to middle cerebral artery (STA-MCA) operations (MMD-O) was significantly lower than that in the PBMNCs cultured from controls (8.70/dish)(p < 0.01). There was no difference in the number of colony-forming definitive cells between patients with MMD-O and those who did not (Fig. 2).

**Figure 3:**
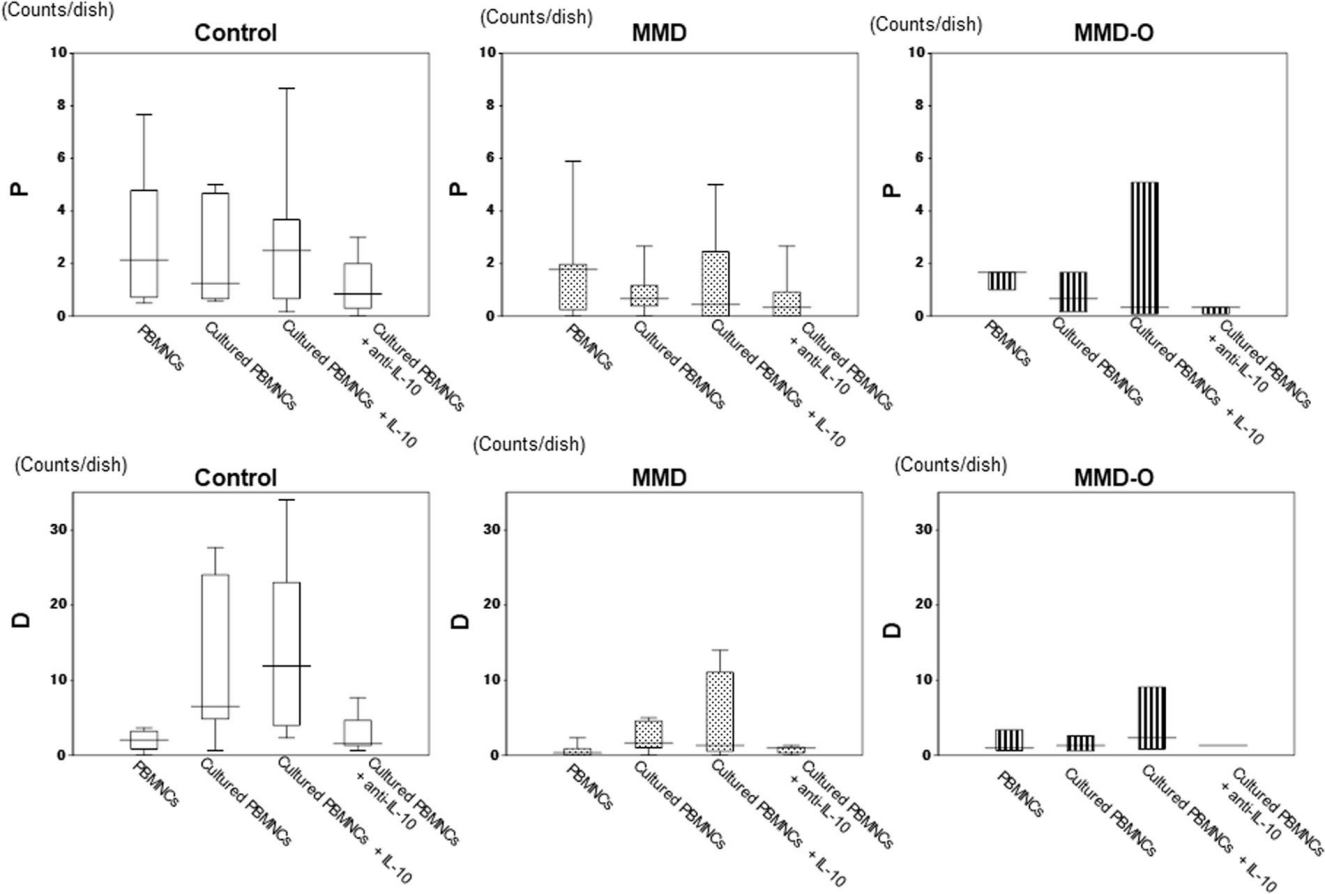
The addition of IL-10 did not alter the number of definitive EPCs in the PBMNCs cultures from patients with MMD. When IL-10 was added to the cultured PBMNCs derived from controls (n = 6), the definitive EPC population increased, however, the same procedure had no effect on the PBMNCs cultured from MMD (n = 7) or MMD-O (n = 3) patients. The number of definitive EPCs decreased in the PBMNCs cultured from controls relative to those from MMD and MMD-O patients when anti-IL-10 antibody was added. With regards to primitive EPCs, there were no significant changes between control group and MMD or MMD-O group. The figure was generated by plotting values using a box-and-whisker plot. P: primitive EPC colonies; D: definitive EPC colonies; count/dish: 35 mm dish was used.

Following the addition of interleukin-10 (IL-10) to the PBMNCs cultured from controls (6.53/dish), the population of definitive cells increased (11.92/dish). However, IL-10 had no significant effect on the definitive cell populations of PBMNCs derived from patients with MMD (1.33/dish) or MMD-O (2.33/dish). Moreover, the number of definitive cells in the PBMNCs cultured from controls decreased (1.56/dish) upon addition of anti-IL-10 antibody, while the number of definitive cells in the PBMNCs cultured from MMD (1.00/dish) and MMD-O (1.33/dish) did not change upon addition of anti-IL-10 antibody (Fig. 3). These results suggest that the EPCs of patients with MMD or MMD-O do not respond to IL-10, suggesting a fundamental abnormality associated with MMD.

### Low concentration of IL-10 secreted by the cultured PBMNCs of patients with MMD

**Figure 4:**
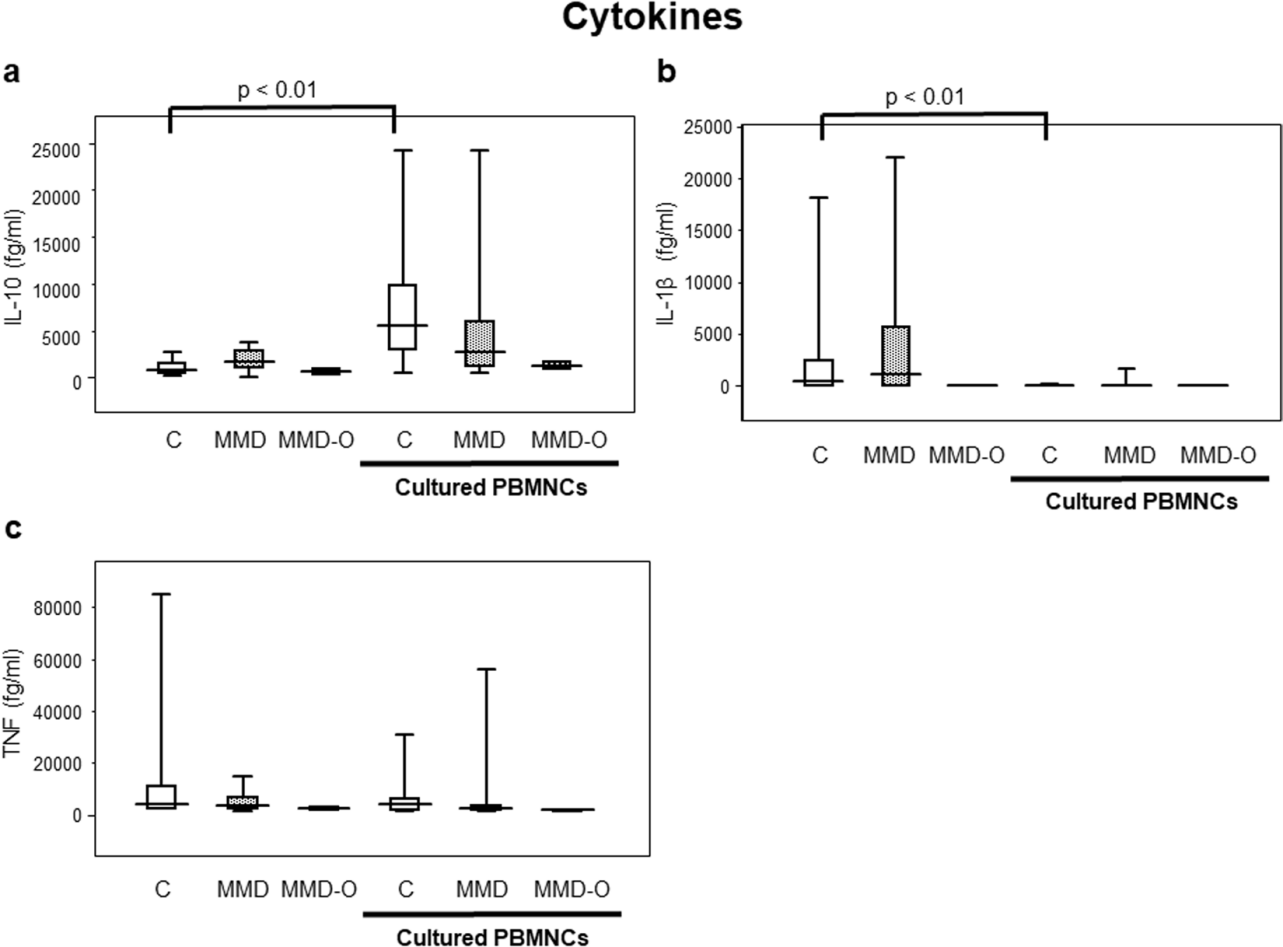
The concentration of secreated cytokines in the freshly isolated PBMNCs and cultured PBMNCs. IL-10 was significantly elevated in the cultured PBMNCs derived from controls (**a**), while IL-1β was decreased in the cultured PBMNCs derived from controls compared to the freshly isolated PBMNCs from controls (**b**). However, the concentration of IL-10 secreted by the PBMNCs cultured from patients with MMD or MMD-O remained stable as compared with the concentration of IL-10 secreted by the PBMNCs from patients with MMD or MMD-O (**a**). The figure was generated by plotting values using a box-and-whisker plot. C, control (n = 23); MMD, Moyamoya disease (n = 23); MMD-O, patients with MMD who underwent superficial temporal artery to middle cerebral artery operations (n = 7); PBMNCs, peripheral blood mononuclear cells.

Using flow cytometry (FCM), we analysed the levels of cytokines in the PBMNCs cultured from patients with MMD or MMD-O and controls to investigate the underlying causes of the above mentioned findings. The concentration of IL-10 secreted by the PBMNCs cultured from controls (5486.84 fg/ml) was significantly higher than that secreted by the PBMNCs derived from controls (868.83 fg/ml) (p < 0.01; Fig. 4a). However, the concentration of IL-10 secreted by the PBMNCs cultured from patients with MMD (2786.12 fg/ml) or MMD-O (1266.69 fg/ml) remained stable as compared with the concentration of IL-10 secreted by the PBMNCs from patients with MMD (1706.24 fg/ml) or MMD-O (626.07 fg/ml) (Fig. 4a).

On the other hand, IL-1β levels secreted by PBMNCs cultured from controls (397.72 fg/ml) were significantly higher than that secreted by PBMNCs from controls (0.00 fg/ml) (p < 0.01). There was no significant difference in the levels of IL-1β between patients with MMD and MMD-O (Fig. 4b).

The alterations of TNF were similar between the freshly isolated PBMNCs and cultured PBMNCs from patients with MMD, MMD-O and controls (Fig. 4c). We could not detect interleukin-4 (IL-4) in freshly isolated PBMNCs and cultured PBMNCs from MMD, MMD-O, and controls.

### The number of M2 macrophages was lower in the PBMNCs from patients with MMD than that in PBMNCs from controls, and the EPCs of patients with MMD were difficult to regenerate in the cultured PBMNCs

**Figure 5:**
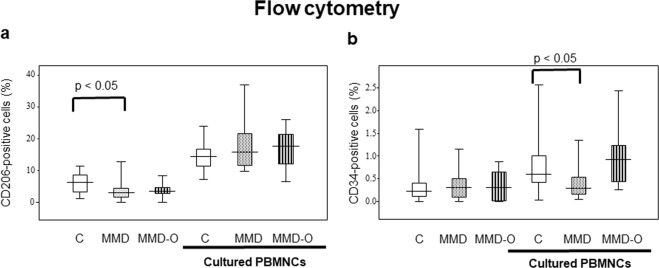
Flow cytometry of MMD patients and controls. The number of CD206 positive cells in the PBMNCs derived from controls was significantly higher than that of patients with MMD (p < 0.05). On the other hand, the number of CD34 positive cells in the PBMNCs cultured from controls was significantly larger than that in the PBMNCs cultured from patients with MMD (p < 0.05). The figure was generated by plotting values using a box-and-whisker plot. C, control; MMD, Moyamoya disease; MMD-O, patients with MMD who underwent superficial temporal artery to middle cerebral artery operations; PBMNCs, peripheral blood mononuclear cells.

Using fluorescence-activated cell sorting (FACS), we discovered that the number of CD206 positive cells in the PBMNCs derived from controls was significantly higher than that of patients with MMD (p < 0.05). No significant differences were observed between the PBMNCs cultured from controls and those cultured from patients with MMD on CD206 positive cells (Fig. 5a). These findings suggest that the number of M2 macrophages was lower in patients with MMD than that in controls.

The number of CD34 positive cells in the PBMNCs cultured from controls was significantly larger than that in the PBMNCs cultured from patients with MMD, indicating decreased growth of EPCs in the PBMNCs derived from patients with MMD relative to those obtained from controls (Fig. 5b).

### The disturbance of IL-10 production in the EPCs of patients with MMD

**Figure 6:**
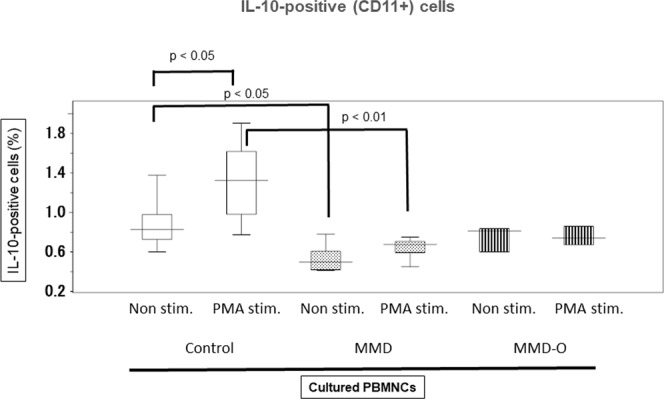
Disruption of IL-10 production in the PBMNCs of patients with MMD. During the non-stimulation phase, the number of IL-10 positive cells in all of the CD11b positive cells of patients with MMD (n = 5) and MMD-O (n = 3) was lower than that of controls (n = 5). During PMA stimulation phase, the number of IL-10 positive cells of controls increased, while those of MMD and MMD-O did not. The figure was generated by plotting values using a box-and-whisker plot. MMD, Moyamoya disease; MMD-O, patients with MMD who underwent superficial temporal artery to middle cerebral artery operations; Non-stim., non-stimulation; PMA stim., phorbol myristate acetate stimulation.

The number of IL-10-positive (CD11b+) PBMNCs from patients with MMD (0.50%) was significantly lower than that from controls (0.83%) during the non-stimulation phase (p < 0.05). Upon stimulation with phorbol myristate acetate (PMA), the number of IL-10-positive (CD11b+) PBMNCs from patients with MMD (0.68%) did not change, but there was a significant increase in the number of IL-10-positive PBMNCs from controls (1.33%)(p < 0.05; Fig. 6). These findings suggest that IL-10 production may be disrupted in the PBMNCs from patients with MMD.

## Discussion

We investigated the characteristics of PBMNCs cultured from patients with MMD, MMD-O under conditions of activated anti-inflammatory and angiogenic monocytes/macrophages. EPCs can participate in normal endothelial repair when a vascular injury occurs, and it is highly plausible that EPCs play a major role in the occlusion of carotid arteries in MMD. In a previous study, it was observed that there were fewer colony-forming units in cells from patients with MMD than in cells from controls, and the number of outgrowth cells was significantly reduced in cells derived from patients with late-stage MMD as compared to outgrowth cells when cells were derived from patients with early-stage MMD^15^.

We have developed the vasculogenic and anti-inflammatory culture system of PBMNCs. The culture system allows to investigate the expansion and differentiation capabilities of endothelial progenitor cells (EPCs) collaborated with the other blood cells, by setting the cytokine milieu for anti-inflammation and angio-vasculogenesis.

In the current study, the angiogenesis in patients with MMD was diminished relative to that of controls; this observation persisted even when angiogenesis was tested in the PBMNCs cultured from patients with MMD (Fig. S1). Moreover, PBMNCs secretions derived from patients with MMD, especially IL-10, reflects poor vasculogenic activity (Fig. S2)^16^.

Besides, the PBMNCs obtained and cultured from patients with MMD did not produce as much IL-10 as the PBMNCs cultured from controls, therefore suggesting a potential role of IL-10 in the pathogenesis of MMD. IL-10 is a cytokine with anti-inflammatory properties, and its loss is associated with autoimmune pathologies^17^. IL-10 is secreted by multiple immune cells, including macrophages, dendritic cells, B cells, and T cells^18^. Our results indicate that the vascular hypoplasia observed in patients with MMD might be caused by IL-10 insufficiency, despite the observed increase in the number of CD206-positive M2 macrophages, which produce and release IL-10 as well as other cytokines and facilitate vasculogenesis^19,20^ in PBMNCs cultured from patients with MMD.

Moreover, similar findings were observed before and after STA-MCA for MMD suggesting IL-10 insufficiency. The current data shows that the EPC expansion and differentiation under the culture of PBMNCs also showed the common tendency between MMD and MMD-O, indicating that the operative treatment did not affect the cellular phenotypes of circulating blood cells. In other words, there might be exist some genetic backgrounds responsible for the abberant cellular phenotypes of circulating blood cells.

Stenosis or occlusion of the cranial division of the internal carotid artery of patients with MMD occurs due to smooth muscle cell proliferation, one of the possible causes of the disease. PDGF, a pro-inflammatory cytokine, is strongly related to the proliferation of smooth muscle cells^21^. Based on our findings, IL-10 insufficiency might induce PDGF activity, and ultimately promote smooth muscle cell proliferation^22^.

Moreover, IL-10 deficiency induces vaascular remodelling in aorta smooth muscle cells via upregulation of Nox1 which is an isoenzyme of gp91box, termed mitogenic oxidase^23^.

IL-10 also improves the function of EPCs stimulated with TNF through the activation of the STAT3 signalling pathway^24^.

Environmental factors may also play a role in triggering pathologic vascular occlusion. Previous reports reveal that RNF213 expression levels increase as the number of IL-10 receptors decreases^25^. Thus, the pathophysiology of MMD may be related to IL-10.

It is still unclear as to why only the cranial segment of the internal carotid artery is affected by stenosis or occlusion in MMD. Although this is a limitation of our study, we propose it might be related to the fact that the internal carotid artery develops as a combination of different segments of the carotid artery^26^.

In conclusion, the observed insufficiency of IL-10 production by blood cells in an anti-inflammatory vasculogenic environment indicates that impaired EPC differentiation may yield an abnormal vascular phenotype critical to the pathogenesis of MMD.

## Materials and Methods

### Participants

All patients with MMD were diagnosed using the criteria established in 2018 by the Research Committee on Spontaneous Occlusion of the Circle of Willis (Moyamoya Disease) Research on Intractable Diseases of the Japanese Ministry of Health, Labor and Welfare^1^. A total of 23 patients with MMD (12 women; mean age, 45 ± 11) who did not undergo an STA-MCA operations, 23 age-matched healthy controls (11 women; mean age, 43 ± 13), and seven patients with MMD-O (5 women; mean age, 39 ± 13) participated in the present study. Patients with MMD who were either under 15 years of age or those with any other disease, such as collagen diseases, Down syndrome, etc., were excluded from participation. Patients characteristics are presented in Supplemental Table S1. All participants were required to provide written informed consent.

Experiments using human samples were performed with institutional approval and according to the guidelines of the Clinical Investigation Committee at Tokai University School of Medicine (Institutional review board approval no. 17R053).

### Cell culture system

Peripheral blood samples were collected from healthy controls and patients with MMD or MMD-O. Histopaque (d = 1.083; Sigma Aldrich, St Louis, Missouri, USA) was used to perform density gradient centrifugation and isolate PBMNCs. Using the quality and quantity of EPCs cultures cell density of 2 × 10^6^ cells/2 mL was obtained and plated into a 6-well Primaria tissue culture plate (BD Falcon; BD Biosciences, New Jersey, USA). Cells were cultured in a defined serum-free medium (StemLineII; Sigma Aldrich, USA), containing five human recombinant proteins (stem cell factor, thrombopoietin, Flt-3 ligand, VEGF, and IL-6) as reported previously^10,11^. Post 7 days, samples were washed with phosphate-buffered solution (PBS) to remove non-attaching cells, and adherent cells were harvested with 2 mmol/L of ethylenediamine tetraacetic acid/phosphate buffered saline (EDTA/PBS). Harvested cells were suspended in Iscove’s modified Dulbecco’s medium (Sigma Aldrich, MO, USA) at a density of 2 × 10^5^ cells/ 100 µL.

### EPC-colony forming assay (EPC-CFA)

To determine the angiogenic potential of PBMNCs, cells derived from patients with MMD (n = 23), patients with MMD-O (n = 7), and controls (n = 23) were cultured in semisolid medium (MethoCult SFBIT; STEMCELL Technologies Inc., Vancouver, British Columbia, Canada) with provasculogenic growth factors/cytokines on 35-mm Primaria dishes (BD Falcon, USA) as reported previously^10,11^. EPC colonies that adhered to the dishes were then counted^9^. Aliquots of the cells were seeded at 2 × 10^5^ cells/dish (three dishes per PBMNCs/cultured PBMNCs) for the EPC-CFA. The number of adherent colonies per dish was measured using a gridded scoring dish (STEM CELL Technologies, Canada) under a phase-contrast light microscope (Eclipse TE300; Nikon, Tokyo, Japan) 16 to 20 days after the initiation of the culture.

We measured the numbers of small- and large-cell colonies per dish after IL-10 (Pepro Tech, Inc., TX, USA) or anti-IL-10 (Abcam, UK) antibody was added to the freshly isolated PBMNCs or those cultured from patients with MMD (n = 7), patients with MMD-O (n = 3) and controls (n = 6).

### Flow cytometry (FCM) for lineage cell populations and cytokines

Flow cytometry was performed to determine the expression of hematopoietic stem cell, lineage-committed cell, and endothelial cell markers on freshly isolated PBMNCs and cultured PBMNCs derived from patients with MMD (n = 23), patients with MMD-O (n = 7), and controls (n = 23). Following antibodies were used: CD206, CD34, and CD11b (BioLegend, CA, USA). Cells were suspended in 2 mmol/L of EDTA/0.2% bovine serum albumin (BSA)/PBS buffer (5 × 10^5^ cells/200 μL) and incubated at 4 °C for 30 minutes following the addition of 10 μL FcR blocking reagent. Next, the suspended cells were equally dispensed into reaction tubes for subsequent staining (100 μL/tube). Each suspended cell was incubated with 2 μL of each antibody at 4 °C for 20 minutes, washed twice with 1 mL of 2 mmol/L EDTA/0.2% BSA/PBS buffer, and suspended in 2 mmol/L of EDTA/0.2% BSA/PBS buffer (2 × 10^5^ cells/200 μL).

The concentrations of IL-10, TNF, IL-1β, and IL-4 were measured in supernatants of freshly isolated and cultured PBMNCs from patients with MMD, patients with MMD-O, and controls using IL-10, TNF, IL-1β, and IL-4 antibodies (BD cytometric bead array human enhanced sensitivity flex sets, NJ, USA) by FCM.

FCM analysis was performed using the LSRFortessa cell analyser (BD Biosciences) and FlowJo software (Tomy Digital Biology Co., Ltd., Tokyo, Japan).

### Stimulation of the cultured PBMNCs with PMA/ionomycin

PBMNCs cultured from MMD (n = 5), MMD-O (n = 3), and control patients (n = 5) were stimulated for 2 h with PMA (10 ng/μL, Sigma Aldrich, MO, USA) and ionomycin (1 μg/μL, Sigma Aldrich, MO, USA) in monensin (1 μl/μL) (BD Biosciences) or brefeldin A (5 μg/mL) (Sigma Aldrich, MO, USA) and incubated in a humidified chamber (95% air, 5% CO_2_, 37 °C).

After incubation, cells were harvested and measured using FACS according to the aforementioned protocol.

### Statistical analysis

Statistical comparisons were performed with SPSS ver. 25. The Wilcoxon and Mann–Whitney U-test were used for comparisons between two groups. For analyses, p < 0.05 was considered statistically significant. Data are presented as mean ± standard deviation (SD).

The results of the tube formation assay and carotid artery ring assay are presented in the supplementary information.

## Supplementary information

Supplementary information

## Data Availability

All data generated or analysed during this study are included in this article and its supplementary information files.
