# Author Correction: Insufficient production of IL-10 from M2 macrophages impairs *in vitro* endothelial progenitor cell differentiation in patients with Moyamoya disease

**DOI:** 10.1038/s41598-020-59305-8

**Published:** 2020-02-06

**Authors:** Eiichiro Nagata, Haruchika Masuda, Taira Nakayama, Shizuka Netsu, Hiroko Yuzawa, Natsuko Fujii, Saori Kohara, Takatoshi Sorimachi, Takahiro Osada, Ryoko Imazeki, Mitsunori Matsumae, Takayuki Asahara, Shunya Takizawa

**Affiliations:** 1 Department of Neurology, Tokai University School of Medicine, Isehara, Japan; 2 Department of Physiology, Tokai University School of Medicine, Isehara, Japan; 3 Department of Neurosurgery, Tokai University School of Medicine, Isehara, Japan; 4 Department of Basic Clinical Science, Division of Regenerative Medicine, Tokai University School of Medicine, Isehara, Japan

Correction to: *Scientific Reports* 10.1038/s41598-019-53114-4, published online 14 November 2019

In this Article the authors did not make it clear that the research was done on early endothelial progenitor cells (CFU-ECs). Two additional articles describing the method of generation of these cells should also be cited. They are included here as Ref 1 and Ref 2. Therefore, in the Introduction,

“This study investigates the relationship between expansion and differentiation ability of EPCs and co-existing monocyte/macrophage-produced cytokines in patients with MMD by using an anti-inflammatory and vasculogenic culture milieu^9,10^.”

should read:

“This study investigates the relationship between expansion and differentiation ability of hematopoietic EPCs^1,2^ and co-existing monocyte/macrophage-produced cytokines in patients with MMD by using an anti-inflammatory and vasculogenic culture milieu^9,10^.”
