# Supplementary material for: Insufficient production of IL-10 from M2 macrophages impairs in vitro endothelial progenitor cell differentiation in patients with Moyamoya disease
Source: Sci Rep. 2019 Nov 14;9:16752. doi: 10.1038/s41598-019-53114-4 (PMC6856135; doi:10.1038/s41598-019-53114-4)
Supplement: Supplementary file 1 — Supplementary information [file 41598_2019_53114_MOESM1_ESM.pdf]

## Supplementary information

**Insufficient production of IL-10 from M2 macrophages impairs *in vitro* endothelial progenitor cell differentiation in patients with Moyamoya disease**

Eiichiro Nagata[1*], Haruchika Masuda[2], Taira Nakayama[1], Shizuka Netsu[1], Hiroko Yuzawa[1], Natsuko Fujii[1], Saori Kohara[1], Takatoshi Sorimachi[3], Takahiro Osada[3], Ryoko Imazeki[1], Mitsunori Matsumae[3], Takayuki Asahara[4], Shunya Takizawa[1]

[1]Department of Neurology, Tokai University School of Medicine, Isehara, Japan

[2]Department of Physiology, Tokai University School of Medicine, Isehara, Japan

[3]Department of Neurosurgery, Tokai University School of Medicine, Isehara, Japan

[4]Department of Basic Clinical Science, Division of Regenerative Medicine, Tokai University School of Medicine, Isehara, Japan

## Supplementary methods

### Tube formation assay in isolated peripheral blood mononuclear cells (PBMNCs) and PBMNCs cultured from patients with Moyamoya Disease (MMD) and healthy controls

Isolated PBMNCs and cultured PBMNCs were resuspended together with human umbilical vein endothelial cells (HUVECs) at the following ratio: endothelial progenitor cells [EPCs]: HUVECs = $3\times10^3$:$1.5\times10^4$ in 100 μL of 1% FBS/EBM-2. The mixed cell suspension was incubated at 37 °C in a water bath and applied at 100 μL each sample onto 50 μL preincubated Matrigel (BD Falcon, USA) in each 96-well plate (BD Falcon; BD Biosciences, USA). After incubation for 6 hours, number of closed areas formed by HUVECs were counted using Photoshop software. The tube and cellular numbers were counted independently by two blinded investigators.

### Carotid artery ring assay

All animal procedures were performed with institutional approval and according to the guidelines of the Experimental Animal Committee at Tokai University School of Medicine (No. 19191).

Carotid artery rings obtained from 1- to 2-month-old enhanced green fluorescence protein (EGFP) male mice (background was C56BL/6N) were cultured in Matrigels (BD Falcon, USA) as previously described.[1] Individually cultured supernatants (50 μl) were put to a 20 μl matrix gel drop in 96-well dishes with a carotid artery ring. The carotid ring was placed at a distance of 0.5 to 1 mm from each well. The plates were incubated at 37 °C in a humidified $CO_2$ incubator for 5 days.

Angiogenesis was shown by the sprouting of blood vessels that grew up from a fragment of the EGFP mouse carotid artery (green colour). The sprouting vessels were counted thrice.

**Table S1. The profiling of patients with Moyamoya Disease (MMD)**

The onset of MMD revealed symptoms associated with transient ischemic attack (TIA) in most cases.

M, male; F, female; ST-MCA operation, superficial temporal artery to middle cerebral artery operations. SAH: subarachnoid haemorrhage

**Figure S1. Tube formation assay in peripheral blood mononuclear cells (PBMNCs) and the cultured PBMNCs of patients with Moyamoya Disease (MMD) and healthy controls[1]**

a, b Bar graphs represent the number of circles counted under a ×2 HPF. The number of circles in the PBMNCs of patients with MMD were significantly smaller than the number of circles in the PBMNCs of healthy controls. HPF, high power field; HUVECs, human umbilical vein endothelial cells; MMD, Moyamoya Disease; PBMNCs, peripheral blood mononuclear cells.

**Figure S2. Carotid artery ring assay**

The extent of sprouting was higher under the cultured condition than under normal condition in controls. In both normal and cultured conditions, the extent of sprouting was lower in MMD patients than in controls.

However, in patients with MMD, the addition of IL-10 induced more sprouting under the culture condition compared to the isolated PBMNCs.

a, b. The sprouting vessels growing from fragments of the carotid arteries of a mouse (green). Sprouting was more extensive in the cultured supernatants than that under normal

conditions ($p < 0.05$). Moreover, the addition of IL-10 facilitated the sprouting from the mouse carotid artery in patients with MMD.

The scale bar is 200 µm. MMD, Moyamoya Disease; IL-10, interleukin-10; PBMNCs, peripheral blood mononuclear cells.

**References**

1. Masuda, H., et al. Vasculogenic conditioning of peripheral blood mononuclear cells promotes endothelial progenitor cell expansion and phenotype transition of anti-inflammatory macrophage and T lymphocyte to cells with regenerative potential. *J. Am. Heart. Assoc.* **3,** e000743; 10.1161/JAHA.113.000743 (2014).

**Table S1.** **Supplementary information**

| | Age at taking blood | Gender | Type of stroke | Neurological symptoms | ST-MCA operation |
|---|---|---|---|---|---|
| MMD01 | 37 | F | TIA | dysarthria | - |
| MMD02 | 64 | F | TIA | headache, right hand muscle weakness | - |
| MMD03 | 47 | M | SAH | headache, consciousness disturbance | - |
| MMD04 | 59 | F | TIA | dysarthria | - |
| MMD05 | 49 | F | TIA | aphasia | - |
| MMD06 | 40 | M | TIA | right hemiparesis, right blindness | - |
| MMD07 | 46 | F | TIA | bilateral upper and lower extremities muscle weakness | - |
| MMD08 | 44 | M | TIA | bilateral lower extremities muscle weakness | - |
| MMD09 | 46 | M | TIA | right hand muscle weakness | - |
| MMD10 | 66 | M | TIA | right leg muscle weakness | - |
| MMD11 | 48 | F | TIA | dysarthria, aphasia | - |
| MMD12 | 56 | M | TIA | left hand dysesthesia | - |
| MMD13 | 31 | F | TIA | right hand muscle weakness | - |
| MMD14 | 46 | F | TIA | dysarthria, left hemiparesis | - |
| MMD15 | 43 | F | TIA | dysarthria, aphasia | - |
| MMD16 | 38 | M | TIA | dysarthria, muscle weakness | - |
| MMD17 | 47 | M | TIA | right hand muscle weakness | - |
| MMD18 | 56 | F | TIA | left hand muscle weakness | - |
| MMD19 | 34 | F | TIA | dysarthria, memory disturbance | - |
| MMD20 | 23 | M | | syncope | - |
| MMD21 | 40 | M | TIA | | - |
| MMD22 | 47 | F | TIA | left hand muscle weakness, syncope | - |
| MMD23 | 20 | M | TIA | left hand dysesthesia, legs muscle weakness | - |
| MMD-O1 | 51 | M | TIA | right hand muscle weakness | + |
| MMD-O2 | 44 | F | TIA | left hand muscle weakness | + |
| MMD-O3 | 48 | F | TIA | left hemiparesis | + |
| MMD-O4 | 15 | M | | headache | + |
| MMD-O5 | 18 | F | TIA | right hemiparesis | + |
| MMD-O6 | 45 | F | TIA | right hand dysesthesia | + |
| MMD-O7 | 52 | F | TIA | right hemiparesis | + |

a

b

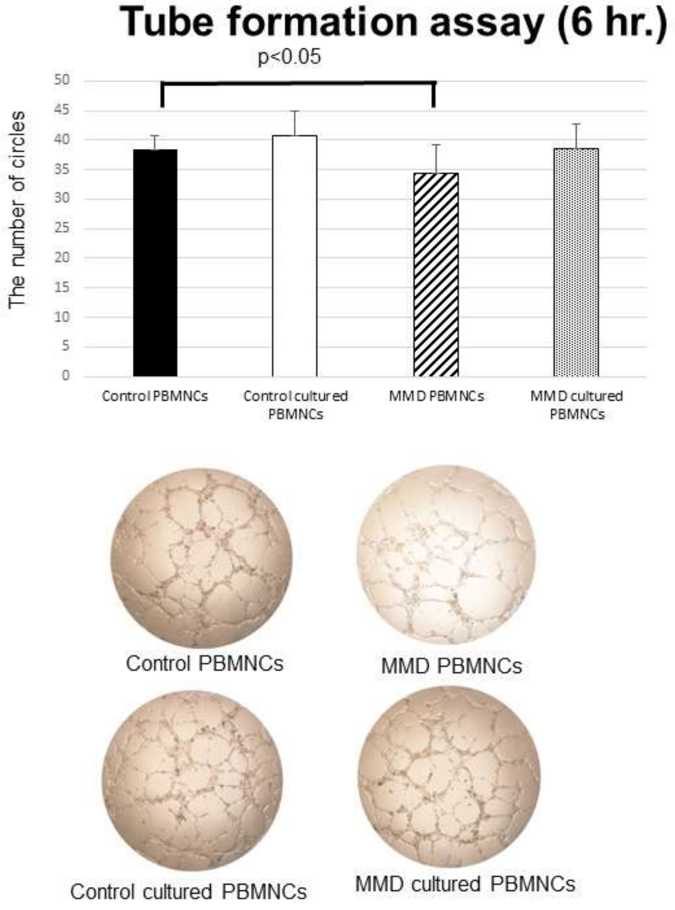
Tube formation assay (6 hr.)
p<0.05
The number of circles
0
5
10
15
20
25
30
35
40
45
50
Control PBMNCs
Control cultured PBMNCs
MMD PBMNCs
MMD cultured PBMNCs
Control PBMNCs
MMD PBMNCs
Control cultured PBMNCs
MMD cultured PBMNCs

**Fig. S 1.**

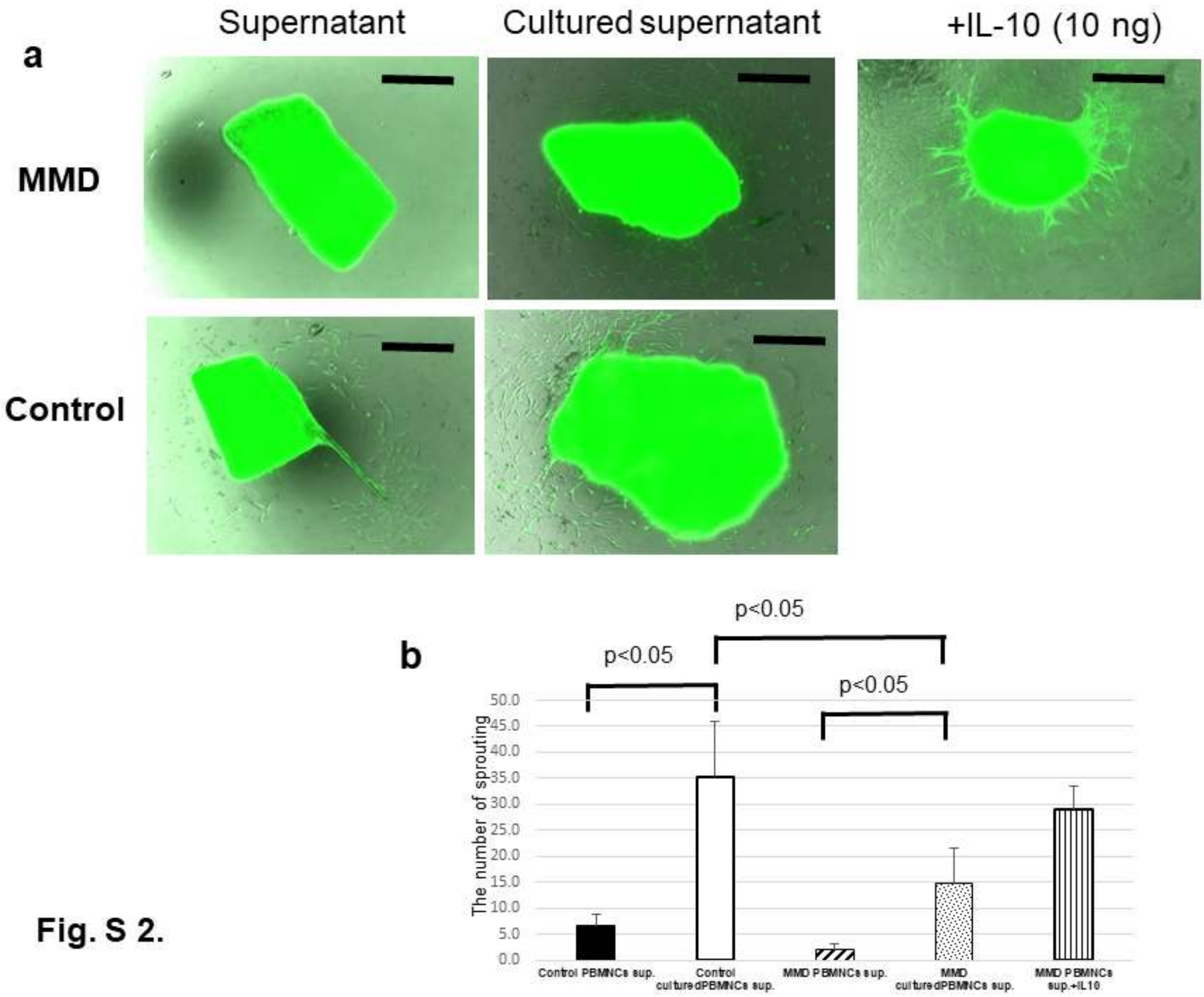
a
Supernatant
Cultured supernatant
+IL-10 (10 ng)
MMD
Control
b
p<0.05
p<0.05
p<0.05
The number of sprouting
50.0
45.0
40.0
35.0
30.0
25.0
20.0
15.0
10.0
5.0
0.0
Control PBMNCs sup.
Control culturedPBMNCs sup.
MMD PBMNCs sup.
MMD culturedPBMNCs sup.
MMD PBMNCs sup.+IL10

**Fig. S 2.**
